# *PlasmiR*: A Manual Collection of Circulating microRNAs of Prognostic and Diagnostic Value

**DOI:** 10.3390/cancers13153680

**Published:** 2021-07-22

**Authors:** Spyros Tastsoglou, Marios Miliotis, Ioannis Kavakiotis, Athanasios Alexiou, Eleni C. Gkotsi, Anastasia Lambropoulou, Vasileios Lygnos, Vasiliki Kotsira, Vasileios Maroulis, Dimitrios Zisis, Giorgos Skoufos, Artemis G. Hatzigeorgiou

**Affiliations:** 1 Hellenic Pasteur Institute, 11521 Athens, Greece; mmiliotis@uth.gr (M.M.); thanosalexiou@uth.gr (A.A.); an.lamprop@pasteur.gr (A.L.); vassilislignos@pasteur.gr (V.L.); dimitris.zisis@pasteur.gr (D.Z.); gskoufos@uth.gr (G.S.); 2 DIANA-Lab, Department of Computer Science and Biomedical Informatics, University of Thessaly, 35131 Lamia, Greece; ikavakiotis@uth.gr (I.K.); vkotsira@uth.gr (V.K.); 3 Department of Informatics and Telecommunications, Postgraduate Program: ‘Information Technologies in Medicine and Biology’, University of Athens, 15784 Athens, Greece; helgkotsi@di.uoa.gr (E.C.G.); maroulisv@biol.uoa.gr (V.M.); 4 Department of Electrical and Computer Engineering, University of Thessaly, 38221 Volos, Greece

**Keywords:** circulating miRNAs, diagnostic miRNAs, prognostic miRNAs, disease biomarker assessment, circulating biomarker collection, circulating biomarker miRNA database

## Abstract

**Simple Summary:**

Only recently have the important biomarker capacities of microRNAs (miRNAs) in blood samples during disease been revealed. miRNAs are abundantly detected in circulation, and are less prone to degradation than longer RNA. Details regarding potential discriminatory miRNAs against numerous pathologic conditions are dispersed across articles, while existing resources that catalogue miRNA abundance in blood samples are not tailored to biomarker research. This study presents the meticulous manual curation of more than 200 articles that specifically interrogate the biomarker potential of miRNAs in whole blood, serum, or plasma. This annotation effort resulted in the creation of *plasmiR*, a database that systematically provides experimental evidence for the diagnostic and prognostic potential of circulating miRNAs against human diseases. *plasmiR* features 1021 entries, accompanied by rich study-specific meta-information, and an intuitive interface that enables the formation of complex queries and visualizations.

**Abstract:**

Only recently, microRNAs (miRNAs) were found to exist in traceable and distinctive amounts in the human circulatory system, bringing forth the intriguing possibility of using them as minimally invasive biomarkers. miRNAs are short non-coding RNAs that act as potent post-transcriptional regulators of gene expression. Extensive studies in cancer and other disease landscapes investigate the protective/pathogenic functions of dysregulated miRNAs, as well as their biomarker potential. A specialized resource amassing experimentally verified, circulating miRNA biomarkers does not exist. We queried the existing literature to identify articles assessing diagnostic/prognostic roles of miRNAs in blood, serum, or plasma samples. Articles were scrutinized in order to exclude instances lacking sufficient experimental documentation or employing no biomarker assessment methods. We incorporated information from more than 200 biomedical articles, annotating crucial meta-information including cohort sizes, inclusion-exclusion criteria, disease/healthy confirmation methods and quantification details. miRNAs and diseases were systematically characterized using reference resources. Our circulating miRNA biomarker collection is provided as an online database, *plasmiR*. It consists of 1021 entries regarding 251 miRNAs and 112 diseases. More than half of *plasmiR*’s entries refer to cancerous and neoplastic conditions, 183 of them (32%) describing prognostic associations. *plasmiR* facilitates smart queries, emphasizing visualization and exploratory modes for all researchers.

## 1. Introduction

microRNAs (miRNAs) are ~22nt non-coding RNAs that associate with the RNA-induced silencing complex (RISC) to guide targeted transcript degradation and/or translational suppression or stall [1]. Most transcripts and, subsequently, biological processes are considered to be under regulation from one or multiple miRNAs, deeming miRNA-mediated transcriptional dysregulation yet another important molecular factor involved in pathogenesis and a field of notoriously active research using specialized experimental and in silico methods [2,3,4].

The protein-bound state of miRNAs and their occasional packaging in vesicles protect them from RNA degradation [5]. In this manner, miRNAs constitute ideal candidate diagnostic and prognostic biomarker RNAs, provided they carry discriminatory capacity.

miRNAs are traceable and differentially abundant in the circulatory system in distinct pathophysiological conditions [6,7]. Blood miRNAs offer the potential to function as minimally invasive biomarkers. A growing body of publications that estimate miRNA abundance in blood and blood derivatives (serum and plasma), investigate miRNAs’ potential to act as diagnostic markers and discriminate between healthy and disease states and to possess prognostic value, correlating significantly with a plethora of disease outcomes including metastasis, relapse, overall, recurrence-free, and post-operative survival [5,8,9].

Quantitative real-time PCR (qRT-PCR), miRNA microarrays and small RNA sequencing (sRNA-Seq) are typically employed for miRNA quantification. Very recently, novel colorimetric [10], electrochemical [11,12], and nanotechnology-based methods [13], as well as molecular/enzymatic assays [14,15] have been proposed. Such approaches could enormously enhance the bench to bedside applicability and value of miRNA biomarkers, by delivering accurate, fast, and potentially inexpensive results, even in settings lacking qRT-PCR instrumentation.

Numerous studies estimate miRNA abundance in blood, serum, and plasma, and conduct differential abundance analyses between patient and healthy groups, or among patient subgroups. Robust biomarker identification efforts go beyond differential abundance analysis, specifically interrogating the value of candidate miRNAs. The most widely applied method to assess diagnostic potential is Receiver Operating Characteristic (ROC) analysis, combined with a criterion to select cut-off points that optimally dichotomize the test values, such as the point maximizing Youden index [16]. Prognostic biomarker assessment studies routinely employ log-rank tests and Kaplan–Meier analysis to evaluate significance in survival between cohort subgroups, univariate, or multivariate Cox regression models to evaluate potential biomarkers and adjust for confounders [17,18]. More sophisticated approaches include the development and evaluation of machine learning models and the combination of multiple miRNAs into multi-component signatures and risk-scores [19,20,21].

A number of databases harboring information on varying scopes of disease-relevant circulating miRNAs currently exist. miRandola features extracellular miRNAs, long non-coding RNAs and circular RNAs that are dysregulated in disease conditions [22]. A number of its entries are manually annotated with biomarker validation method information (“Experiment Description” field). However, as its prime target is to provide a wider spectrum of data, it does not offer biomarker-oriented query options, making this information sparse and difficult to extract. Additionally, metadata regarding the statistical methods and cohorts is missing. The human miRNA-disease association (HMDD) database collects (epi-)genetics, targeting, tissue-expression, and circulatory miRNA-disease associations [23]. The HMDD circulation category emphasizes on the up- or down-regulated status of miRNAs in disease states and also incorporates target-based functional enrichment analysis. The Circulating MicroRNA Expression Profiling (CMEP) database utilizes biofluid expression profiles from available sequencing or microarray experiments to provide an online platform for differential expression, pathway enrichment, and potential diagnostic marker analysis [24].

In this study, we manually curated available articles that use bona fide evaluation methods, as noted above, to assess the diagnostic and/or prognostic roles (i) of miRNAs circulating freely in plasma/serum fractions or, if not otherwise specified, (ii) of miRNAs found in blood samples. Our aim was to create a comprehensive and systematically annotated resource, and provide it in a schema that encourages smart queries, cross-disease contrasts, and hypothesis-free explorations in the circulating miRNA biomarker space. Contrary to existing resources, we focused specifically on bringing to the fore the biomarker validation choices, and experimental and statistical methods that each study has employed, as well as extensive cohort details. Additionally, we attempted to provide rich interactive visualization capacities, as well as seamless interconnection with reference miRNA target databases and user-parameterizable functional annotation resources, to further enhance the usability of our application.

## 2. Materials and Methods

### 2.1. Article Collection

Initially, we searched PubMed and PubMed Central non-systematically, by forming queries using relevant keywords, such as “miRNA”, “biomarker”, “signature”, “diagnostic”, “prognostic”, “circulating”, “blood”, “serum”, and “plasma”. Resulting entries were filtered by inspecting the titles and abstracts to exclude false hits and separate review articles. Articles referenced in reviews were also inspected to keep potentially relevant instances. This process created a primary set of 411 candidate publications (publication years ranging from 2003 to 2021) for curation.

### 2.2. Curation

A template sheet was created to maximize uniform curation of primary data and metadata. The template fields included “disease”, “species”, “miRNA name”, “mature miRNA identifier”, “miRNA abundance in disease” (i.e., up/down), “sample type” (blood, plasma or serum), “quantification method”, “biomarker type” (diagnostic/prognostic), “biomarker assessment methods”, “prognostic outcome”, “association direction”, “PubMed ID”, “article title”, “authors”, “journal name”, “year”, and “curator comment”. Additional fields included healthy and patient cohort sizes, sex ratios, mean/median age, methods to confirm healthy/disease status, treatment information, and quantification details (e.g., utilized kits).

We declared that each database entry would either denote the diagnostic or prognostic value of one miRNA against one disease or prognostic outcome, respectively. Repeated assessments against additional cohorts in the same study were annotated as separate entries to enable correct annotation of the cohort and method meta-information. The same rationale (i.e., separate database entries) was applied for studies concurrently exploring both diagnostic and prognostic role(s) of the same miRNA(s), and for studies evaluating miRNA abundance in more than one sample types (plasma and serum). “disease” and “prognostic outcome” were designated as free-text fields, while “miRNA name” was validated during curation in miRBase v22.1 reference database [25]. If the publication did not discriminate between “-5p” and “-3p” miRNA forms, and did not provide the miRNA sequence, miRBase was queried to match the publication miRNA name with mature miRNA names from previous versions lacking the “5p/3p” nomenclature, or, finally, the most abundant form as annotated in miRBase was selected. The “curator comment” field enabled curators to pinpoint such instances, as well as other potential discrepancies.

After initial curation, database entries were quality controlled by two independent curators and consistency checks were applied across the whole dataset. Validation, resource interconnection, creation of the final database tables, as well as calculation of metrics and creation of the manuscript bar-plots, heatmaps, and the circular plot, were performed using R 3.5.2 [26], and packages data.table [27], ggplot2 [28], pheatmap [29], and circlize [30].

### 2.3. Interconnection with External Reference Resources

miRBase names, identifiers and confidence levels (i.e., “High/Low”) were combined with RNAcentral [31] and MirGeneDB 2.0 [32] identifiers. Systematic disease names and synonyms were retrieved from the Disease Vocabulary of the Comparative Toxicogenomics Database (CTD) [33] and manually matched with the curated diseases. Active hyperlinks pointing to miRBase, RNAcentral, CTD, Medical Subject Headings (MeSH) Disease [34], Disease Ontology [35], Online Mendelian Inheritance in Man (OMIM) [36], PubMed, DIANA-TarBase v8.0 [37] and DIANA-miRPath v3.0 [38] were created.

### 2.4. Database Architecture and Development

An SQL database under MVC architecture was built and hosted on Apache HTTP server 2.4. The back-end was composed of PostgreSQL v12.6 (https://www.postgresql.org/; accessed on 11 February 2021) and the PHP framework Laravel 8 (https://laravel.com/; accessed on 10 October 2020) (PHP 7.2). The front-end was designed using Angular 9.1 (https://angular.io/; accessed on 10 February 2021) and library Angular Material UI (https://material.angular.io/; accessed on 10 February 2021). *plasmiR* data are stored in SQL tables and all connections among them for retrieval are handled by Laravel. The database statistics and result-specific visualizations are presented in the presentation layer using Chart JS (https://www.chartjs.org/; accessed on 14 April 2021) and Plotly JavaScript Open Source Graphing Library (https://plotly.com/javascript/; accessed on 14 April 2021). Flourish application (https://flourish.studio/; accessed on 21 May 2021) is utilized to render the exploratory miRNA-disease network graphs.

## 3. Results

### 3.1. Database Statistics

The curation process yielded 1021 database entries from a total of 204 research articles. *plasmiR* (http://microrna.gr/plasmir/) caters information about 251 circulating miRNAs and 112 systematic disease names. As numerous miRNAs are annotated as biomarkers of more than one disease and vice versa, 594 unique miRNA-disease pairs (i.e., unique combinations of all annotated miRNAs and diseases) are formed (Table 1). Comparable amounts of serum and plasma miRNA biomarkers are offered, while 30% of miRNAs (*n* = 80) feature both diagnostic and prognostic capacities.

The disease landscape covered in *plasmiR* corresponds to 32 cancerous, 26 cardiovascular, 9 neurological, 9 metabolic, and 36 diverse pathological conditions (“Other”). Cancers and neoplasms feature the most entries (*n* = 565) and total miRNAs (*n* = 149), followed by “Other” diseases, which include infection-related entries (e.g., dengue, whooping cough, or sepsis), pregnancy complications (e.g., abruptio placentae or ectopic pregnancy), liver conditions (e.g., hepatitis B, C, acute-on-chronic failure, or cirrhosis), hormone deficiencies, autoimmune diseases, and other conditions. Entry and miRNA summaries are provided per disease category in Table 2.

**Figure 1:**
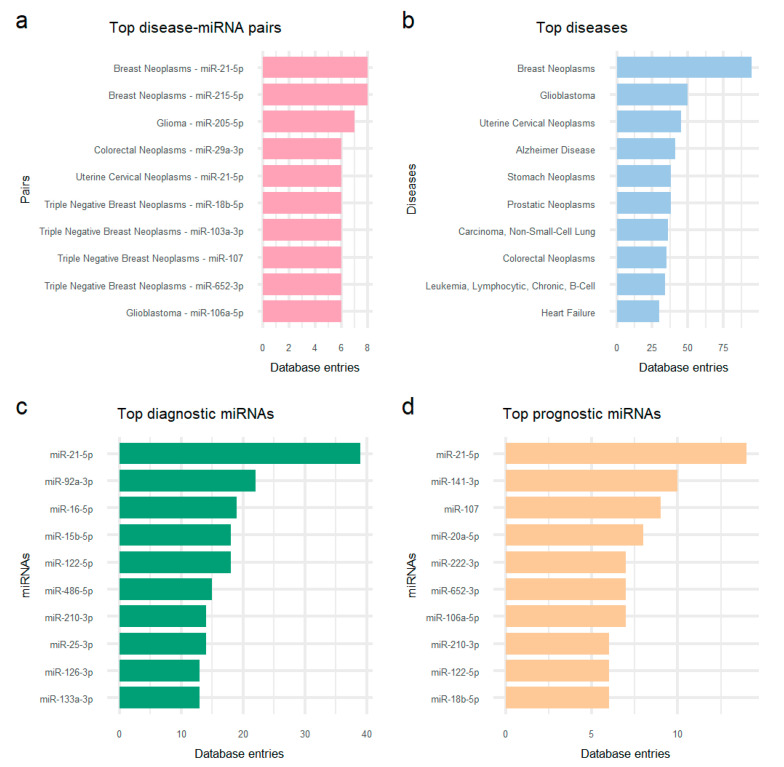
*plasmiR* content. Top 10 (**a**) disease-miRNA pairs, (**b**) systematically annotated diseases, (**c**) diagnostic, and (**d**) prognostic miRNAs, in terms of entry sums.

As shown in Figure 1a, nine miRNAs (i.e., 21-5p, 215-5p, 205-5p, 29a-3p, 18b-5p, 103a-3p, 107, 652-3p, and 106a-5p) and breast, colorectal, cervical, and brain neoplasms are included in the top miRNA-disease pairs supported by the most database entries. The diseases featured the most in *plasmiR* are breast neoplasms (*n* = 95 entries), glioblastoma (*n* = 50), cervical neoplasms (*n* = 45), Alzheimer’s disease (*n* = 41), stomach (*n* = 38) and prostatic neoplasms (*n* = 38), non-small-cell lung carcinoma (*n* = 36), colorectal neoplasms (*n* = 35), chronic lymphocytic B-cell leukemia (*n* = 34), and heart failure (*n* = 30) (Figure 1b). The top represented circulating miRNAs with experimental evidence (Figure 1c,d) included top diagnostic and top prognostic miRNAs 21-5p (*n*_diagnostic_ = 39, *n*_prognostic_ = 14), 122-5p (*n*_diagnostic_ = 18, *n*_prognostic_ = 6), and 210-3p (*n*_diagnostic_ = 14, *n*_prognostic_ = 6), top diagnostic miRNAs 92a-3p (*n* = 22), 16-5p (*n* = 19), 15b-5p (*n* = 18), 486-5p (*n* = 15), 25-3p (*n* = 14), 126-3p (*n* = 13), and 133a-3p (*n* = 13), and top prognostic miRNAs 141-3p (*n* = 10), 107 (*n* = 9), 20a-5p (*n* = 8), 222-3p (*n* = 7), 652-3p (*n* = 7), 106a-5p (*n* = 7), and 18b-5p (*n* = 6).

**Figure 2:**
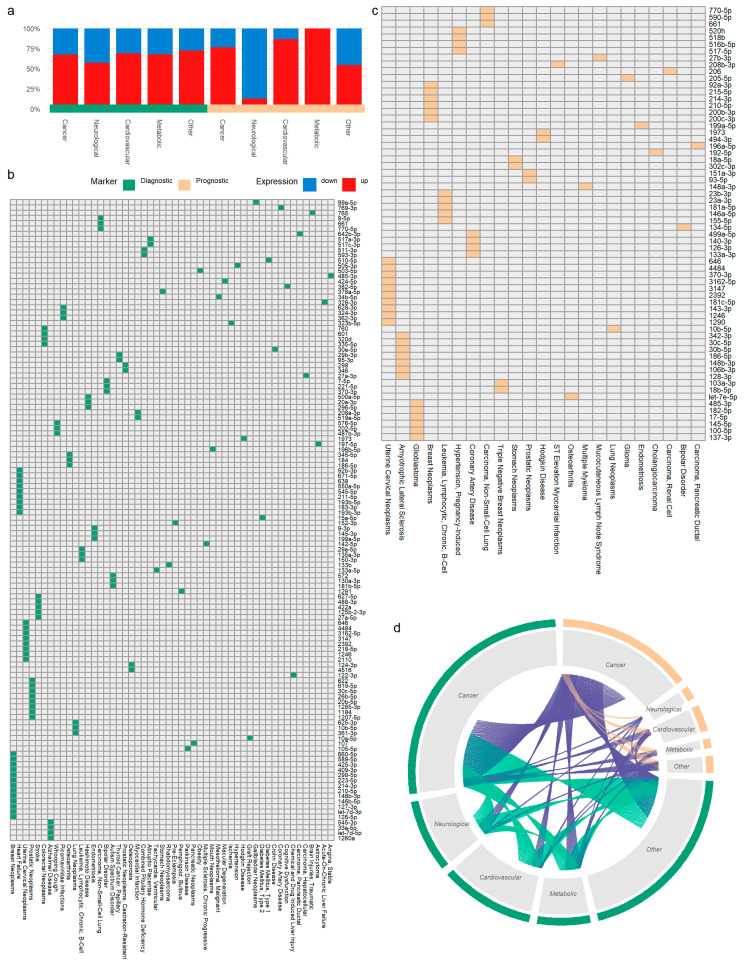
Composite view of the *plasmiR* content. (**a**) Percentage of up- (red) and down-regulated (blue) miRNAs in each disease category for diagnostic (green) and prognostic (light orange) entries. (**b**) Heatmap presenting 122 miRNAs annotated as diagnostic biomarkers against a single disease each (52 diseases). (**c**) Heatmap of 64 prognostic miRNAs against outcomes of 23 single diseases. (**d**) Entries are grouped by biomarker type (outer arcs, green: diagnostic, light orange: prognostic) and per disease category (inner grey arcs, cancers, neurological conditions, cardiovascular diseases, metabolic disorders, and other diseases). In the center of the plot, the number of strings that link distinct disease arcs matches the amounts of shared miRNAs across disease categories that are found to possess biomarker potential (green strings: diagnostic-diagnostic links, orange: prognostic-prognostic links, and purple: diagnostic-prognostic links).

With the exception of neurological disorders, the majority of potential biomarker miRNAs are found up-regulated in the studied disease states, with regard to healthy controls (Figure 2a). Within disease categories, most miRNAs feature a relatively narrow window of biomarker potential, spanning one or few diseases. Specifically, regarding diagnostic entries, 77%, 90%, 80%, 90%, and 96% of miRNAs in cancer, neurological, cardiovascular, metabolic, and “Other” disease categories, respectively, have up to three biomarker entries per miRNA. The same applies for 79% of miRNAs with prognostic capacity in cancers. Notably, 122 diagnostic and 64 prognostic miRNAs solely present a validated biomarker role against one single disease each (diagnostic relationships for 52 diseases and prognostic associations for 23 diseases, respectively, Figure 2b,c).

On the other hand, numerous miRNAs within cancers appear with both diagnostic and prognostic roles (42% of diagnostic cancer miRNAs; purple strings linking cancers in Figure 2d), while an overlap of miRNAs across disease categories can also be observed (e.g., 19 diagnostic miRNAs overlap between cancers and cardiovascular diseases; green strings in Figure 2d).

Regarding applied methods to assess biomarker value, 623 diagnostic entries in *plasmiR* (78%) are validated using ROC analysis. A total of 145 prognostic entries (64%) are derived from articles employing the log-rank test and/or Kaplan–Meier analysis and/or odds ratios and/or Cox Regression analysis (univariate/multivariate). Out of 225 entries assessing prognostic value, 113 (50%) refer to patient survival (overall, post-operative, disease-, metastasis-, recurrence-, progression-, and treatment-free).

### 3.2. Database Functionality

Queries in *plasmiR* are formed mainly via providing one, multiple, or all mature miRNA names and/or systematic disease names. A number of filtering options are available, including miRNA expression direction, sample/biomarker type, cohort age range, and minimum accepted cohort size. Drop-down menus have been implemented to eliminate the chance of mistyped queries.

**Figure 3:**
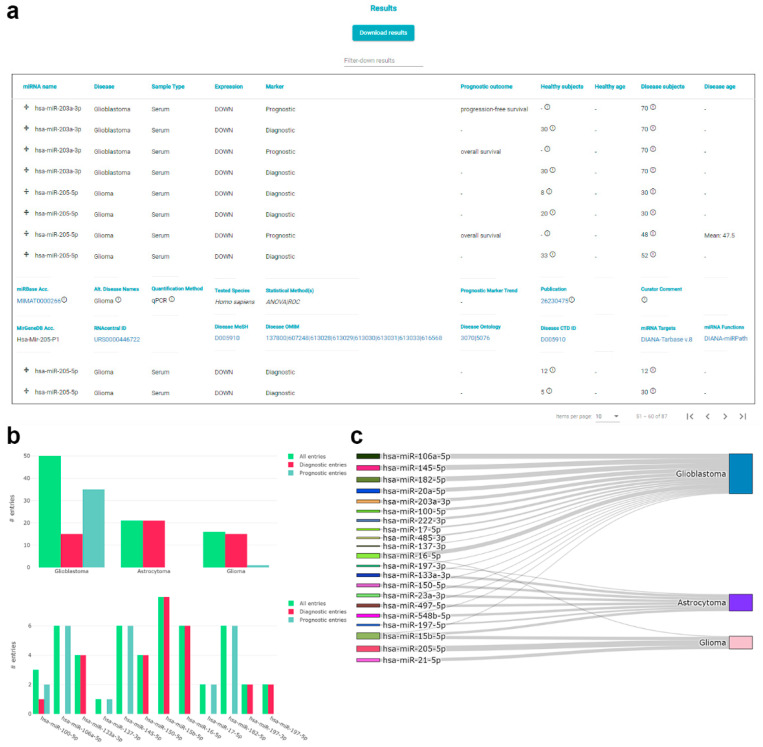
Screenshots of the *plasmiR* results interface. (**a**) Results are provided in a coherent, paged table format. The main information of each entry includes the mature miRNA name, the systematic disease name, sample type, expression in disease and biomarker type, prognostic outcome, healthy and disease cohort size, and mean or median age. Users may hover over available information buttons (i), or click on a query of interest to reveal further details (e.g., disease name used in the original publication, applied quantification, and statistical methods, publication information) and interconnection with reference resources (miRBase, RNAcentral, MeSH, OMIM, Disease Ontology, CTD, DIANA-TarBase, and DIANA-miRPath). The option to download the results table or narrow it down using keywords is available in the top controls. (**b**) Supplemental bar-plots providing entry numbers of the results table per disease and per miRNA. All/Diagnostic/Prognostic groups can be (de-)activated at any time. Options to zoom-in and -out and retrieve the plots locally in PNG format are offered via a minimal control section in each plot (e.g., depicted plots in (**b**,**c**) were created directly through *plasmiR*’s interface). (**c**) Supplemental Sankey plot showing the relationship between all miRNAs and diseases in the results table. Hovering over points in all plots reveals the numbers of specific miRNA-disease combinations.

The main information provided in *plasmiR* entries is the miRNA-disease pair, the sample type (i.e., plasma, serum, or blood), the biomarker type and assessed outcome in the case of prognostic entries and cohort details (Figure 3a). Users may expand the view on entries of interest to reveal extensive metadata, including experimental and statistical details, the relevant publication, and the curator comments, as well as interconnections to external resources. Links towards DIANA-TarBase and DIANA-miRPath allow on-the-spot browsing of experimentally supported miRNA targets and in silico functional analysis of specific miRNAs. Downstream functional analysis could prove to be especially useful in cases where changes in the abundance of circulating miRNAs could be attributed to disease-relevant events, for example, due to tissue injury or tumor invasiveness and metastasis. In order to further facilitate downstream analyses, apart from direct links to other online resources, the option to retrieve query results locally in tab-delimited format is also provided.

Each query is supplemented with result-specific plots that can be stored locally. Specifically, entries are grouped in two separate bar-plots, per disease and per miRNA (Figure 3b), while a Sankey plot (Figure 3c) is utilized to visualize relationships between biomarkers and diseases (e.g., miRNAs annotated as biomarkers in more than one disease vs. unitary miRNA-disease pairs).

Separate pages in *plasmiR* (*Statistics* and *Visualizations* pages) provide database-wide aspects of the content and can guide specific queries. In *Statistics*, the top miRNAs and diseases, in terms of absolute diagnostic and prognostic entries, are depicted as ordered horizontal bar-plots. In the *Visualizations* page, four interactive network graphs for the main disease types annotated in *plasmiR* are provided (i.e., cancers, neurological, cardiovascular, and metabolic conditions). miRNAs and diseases are presented as nodes; miRNAs are color-coded to denote whether they are annotated as having diagnostic and/or prognostic potential. A help section has been created, describing every component in *plasmiR* resource, and facilitating navigation through its content.

## 4. Discussion

Circulating miRNAs constitute an intriguing part of the disease biomarker field. Their investigation often extends above and beyond the signature space, due to their potent regulatory roles and the means of transportation across tissues and cells, towards the identification of intercellular communication phenomena with oncogenic [39] or protective consequence [40]. Elucidating the specific reasons for which miRNAs could end up significantly dysregulated in blood samples can be challenging. Besides technical hemolysis [41,42], miRNA abundance in blood could be attributed to a number of disease-specific reasons, including infiltrative tumor biology [43], tissue injury [44], the budding of extracellular vesicles into circulation [45], and the induction of system-wide changes affecting miRNA biogenesis in blood cells themselves [46,47]. Dissecting the biological effects and roles of blood miRNAs is the next step. At the same time, the need for cautious interpretation of quantification results is underlined; experimental issues [42], analytical choices [48,49] and potential confounding factors, such as age [46], sex [50], and exercise and dietary habits [51,52], need to be handled appropriately and tracked.

Via *plasmiR*, users can retrieve rich details for their miRNA(s)/disease(s) of interest, and even access wide-aspect views of the currently explored landscape of circulating disease miRNAs. Importantly, they can apply filters to limit the content to specific subsets (e.g., a specific range of mean ages). Via use of the extensive provided plotting capacities, they may pinpoint miRNAs annotated as biomarkers against multiple diseases, or identify singleton miRNA biomarkers discriminating close diseases, and form/support novel hypotheses regarding potential shared traits between pathologic conditions.

We must acknowledge a number of limitations that exist in our study. The quality of existing miRNA annotations is still under debate, even in humans, with both primary miRNA annotation resources, miRBase and MirGeneDB, placing effort towards refining their records via application of abundance-, structure-, and conservation-based strategies [25,32]. By utilizing the latest miRBase version, we were able to remove some miRNA instances that are deemed as false positives (“dead entries”), however the possibility of false positive annotations still exists in miRNA research. Another potential pitfall lies with the use of low-yield techniques, such as qRT-PCR, to verify the value of a small select set of miRNAs. This practice can introduce bias, focusing biomarker research and creating trends towards the most well-studied miRNAs (e.g., miR-21-5p), while other, less noted, candidates could potentially exhibit higher cross-disease specificity. Missing data and reporting bias constitute additional limitations inherited in *plasmiR* from the curated articles. Mean or median cohort age, sex ratios, and cohort sizes were not always available in publications. Notably, our curation effort was dedicated to the cataloguing of positive results; articles reporting on the unsuitability of specific miRNAs to function as biomarkers were scarce and have not been included. Lastly, the possibility exists that our article collection procedure has omitted a number of relevant publications.

The advent of sRNA-Seq and the constant formation of sequencing-based, multi-institute collaborative efforts, such as the exRNA Atlas [53], warrant that available circulating miRNA biomarkers will soon increase even more. A future direction for *plasmiR* is the from-scratch analysis of thousands of publicly available sRNA-Seq datasets from blood derivative samples, to assess the diagnostic and prognostic value of each annotated miRNA against available conditions. High-throughput, hypothesis-free approaches will diminish potential publication and reporting biases that could result in technical enrichment of specific miRNAs in entries derived from assessments using low-yield techniques. These approaches will also yield sets of robust negative results and make possible the comparison of findings across distinct applied methodologies. The expected increase in database entries and sources will enable us to experiment on assigning confidence scores to each potential biomarker and pinpoint discrepancies that might arise. However, the article search and manual curation will not cease. It is in our future plans to utilize the collected article set to implement a specialized text-mining application to aid the literature curation process. In future versions, article retraction queries and discrepancy checks will also be applied to existing database entries, which will be tracked and annotated appropriately.

We also aspire that the process we followed in *plasmiR* could serve as a point of reference for other database creation endeavors which rely on manual curation, in biomedical sciences and other fields. Depending on the relationships between the core data, the wealth of existing literature or other sources, and the designated uses, researchers could possibly benefit by our adopted curation protocol, database field structure, and stated limitations, particularly within the fields of biomarkers, performance evaluation, and time-to-event analysis.

## 5. Conclusions

We provide to the scientific community *plasmiR*, a database of circulating miRNA biomarkers with experimental support, emphasizing the applied methodologies and study details of each entry. We are certain that *plasmiR* will facilitate future validation efforts of specific biomarkers of interest, and that its interface innovations will fuel hypothesis creation and cross-disease comparative investigations of miRNA biomarker potential, especially in the cancer landscape.

## Figures and Tables

**Table 1:** Content metrics for *plasmiR*. Database entry numbers are provided per sample type (Serum/Plasma/Blood) and for the whole database. Sums of entries are further broken down into diagnostic, prognostic, and total (i.e., diagnostic plus prognostic) categories. The sum of unique miRNA-disease pairs is also provided. Numbers of miRNAs that participate in diagnostic, prognostic, or common (i.e., diagnostic and prognostic) pairs are also presented for the whole database and per sample type.

Entry Type	Database Entries			miRNAs		
	Total	Diagnostic	Prognostic	Diagnostic Only	Common	Prognostic Only
Database	1021	796 (78%)	225 (22%)	157	80	14
Serum	499	363 (73%)	136 (27%)	103	49	11
Plasma	475	410 (86%)	65 (14%)	111	34	9
Blood	47	23 (49%)	24 (51%)	15	1	16
Unique miRNA-disease pairs	594	522	184 ^1^	-	-	-

^1^ Refers to unique miRNA-disease-outcome triplets.

**Table 2:** Per disease category content metrics. Entry (total/diagnostic/prognostic) and miRNA numbers (only diagnostic/only prognostic/diagnostic and prognostic) are presented.

Disease Category	Database Entries			miRNAs		
	Total	Diagnostic	Prognostic	Diagnostic Only	Common	Prognostic Only
Cancers-neoplasms	565	382 (68%)	183 (32%)	77	56	16
Cardiovascular	137	121 (88%)	16 (12%)	42	8	7
Neurological-neurodegenerative	90	82 (91%)	8 (9%)	37	2	6
Metabolic	49	43 (88%)	6 (12%)	25	4	2
Other	180	168 (93%)	12 (7%)	93	7	3

## Data Availability

*plasmiR* is available freely and without login requirements online and for local retrieval at http://microrna.gr/plasmir/.

## References

[1] Bartel D.P. (2004). MicroRNAs: Genomics, biogenesis, mechanism, and function. Cell.

[2] Thomson D., Bracken C.P., Goodall G.J. (2011). Experimental strategies for microRNA target identification. Nucleic Acids Res..

[3] Hafner M., Landthaler M., Burger L., Khorshid M., Hausser J., Berninger P., Rothballer A., Ascano M., Jungkamp A.-C., Munschauer M. (2010). Transcriptome-wide identification of RNA-binding protein and microRNA target sites by PAR-CLIP. Cell.

[4] Vlachos I.S., Hatzigeorgiou A.G. (2013). Online resources for miRNA analysis. Clin. Biochem..

[5] Condrat C.E., Thompson D.C., Barbu M.G., Bugnar O.L., Boboc A., Cretoiu D., Suciu N., Cretoiu S.M., Voinea S.C. (2020). MiRNAs as biomarkers in disease: Latest findings regarding their role in diagnosis and prognosis. Cells.

[6] Chim S., Shing T.K.F., Hung E.C.W., Leung T.-Y., Lau T.K., Chiu R.W.K., Lo Y.M.D. (2008). Detection and characterization of placental microRNAs in maternal plasma. Clin. Chem..

[7] Lawrie C.H., Gal S., Dunlop H.M., Pushkaran B., Liggins A.P., Pulford K., Banham A., Pezzella F., Boultwood J., Wainscoat J.S. (2008). Detection of elevated levels of tumour-associated microRNAs in serum of patients with diffuse large B-cell lymphoma. Br. J. Haematol..

[8] Corsten M.F., Dennert R., Jochems S., Kuznetsova T., Devaux Y., Hofstra L., Wagner D.R., Staessen J.A., Heymans S., Schroen B. (2010). Circulating microRNA-208b and microRNA-499 reflect myocardial damage in cardiovascular disease. Circ. Cardiovasc. Genet..

[9] Wiedrick J.T., Phillips J.I., Lusardi T., McFarland T.J., Lind B., Sandau U.S., Harrington C.A., Lapidus J.A., Galasko D.R., Quinn J.F. (2019). Validation of microRNA biomarkers for Alzheimer’s disease in human cerebrospinal fluid. J. Alzheimer Dis..

[10] Fakhri N., Abarghoei S., Dadmehr M., Hosseini M., Sabahi H., Ganjali M.R. (2020). Paper based colorimetric detection of miRNA-21 using Ag/Pt nanoclusters. Spectrochim. Acta Part A Mol. Biomol. Spectrosc..

[11] Low S.S., Pan Y., Ji D., Li Y., Lu Y., He Y., Chen Q., Liu Q. (2020). Smartphone-based portable electrochemical biosensing system for detection of circulating microRNA-21 in saliva as a proof-of-concept. Sens. Actuators B Chem..

[12] Bo B., Zhang T., Jiang Y., Cui H., Miao P. (2018). Triple signal amplification strategy for ultrasensitive determination of miRNA based on duplex specific nuclease and bridge DNA-gold nanoparticles. Anal. Chem..

[13] Chandrasekaran A.R., Punnoose J.A., Zhou L., Dey P., Dey B.K., Halvorsen K. (2019). DNA nanotechnology approaches for microRNA detection and diagnosis. Nucleic Acids Res..

[14] Lim J., Byun J., Guk K., Hwang S.G., Bae P.K., Jung J., Kang T., Lim E.-K. (2019). Highly sensitive in vitro diagnostic system of pandemic influenza A (H1N1) virus infection with specific microRNA as a biomarker. ACS Omega.

[15] Li Q., Zhou S., Zhang T., Zheng B., Tang H. (2020). Bioinspired sensor chip for detection of miRNA-21 based on photonic crystals assisted cyclic enzymatic amplification method. Biosens. Bioelectron..

[16] Habibzadeh F., Habibzadeh P., Yadollahie M. (2016). On determining the most appropriate test cut-off value: The case of tests with continuous results. Biochem. Med..

[17] Clark T., Bradburn M., Love S.B., Altman D.G. (2003). Survival analysis part I: Basic concepts and first analyses. Br. J. Cancer.

[18] Bradburn M.J., Clark T.G., Love S.B., Altman D.G. (2003). Survival analysis part II: Multivariate data analysis—An introduction to concepts and methods. Br. J. Cancer.

[19] Zhao H., Shen J., Hodges T.R., Song R., Fuller G.N., Heimberger A.B. (2017). Serum microRNA profiling in patients with glioblastoma: A survival analysis. Mol. Cancer.

[20] Zhao L., Zhou X., Shan X., Qi L.-W., Wang T., Zhu J., Zhu D., Huang Z., Zhang L., Zhang H. (2018). Differential expression levels of plasma microRNA in Hashimoto’s disease. Gene.

[21] Fu L., Peng Q. (2017). A deep ensemble model to predict miRNA-disease association. Sci. Rep..

[22] Russo F., Di Bella S., Vannini F., Berti G., Scoyni F., Cook H.V., Santos A., Nigita G., Bonnici V., Laganà A. (2017). MiRandola 2017: A curated knowledge base of non-invasive biomarkers. Nucleic Acids Res..

[23] Huang Z., Shi J., Gao Y., Cui C., Zhang S., Li J., Zhou Y., Cui Q. (2019). HMDD v3.0: A database for experimentally supported human microRNA-disease associations. Nucleic Acids Res..

[24] Li J.-R., Tong C.Y., Sung T.-J., Kang T.-Y., Zhou X.J., Liu C.-C. (2019). CMEP: A database for circulating microRNA expression profiling. Bioinformatics.

[25] Kozomara A., Birgaoanu M., Griffiths-Jones S. (2018). MiRBase: From microRNA sequences to function. Nucleic Acids Res..

[26] The R Project for Statistical Computing (2018). R: A Language and Environment for Statistical Computing.

[27] Dowle M., Srinivasan A., Gorecki J., Chirico M., Stetsenko P., Short T., Lianoglou S., Antonyan E., Bonsch M., Parsonage H. (2019). Package “Data.Table”. Extension of “Data.Frame”. https://cran.r-project.org/web/packages/data.table/.

[28] Wickham H. (2011). Ggplot2. Wiley Interdiscip. Rev. Comput. Stat..

[29] Kolde R. (2019). *Pheatmap: Pretty Heatmaps*, version 1.0.12. https://https://cran.r-project.org/web/packages/pheatmap/.

[30] Gu Z., Gu L., Eils R., Schlesner M., Brors B. (2014). Circlize implements and enhances circular visualization in R. Bioinformatics.

[31] Sweeney B.A., RNAcentral Consortium (2019). RNAcentral: A hub of information for non-coding RNA sequences. Nucleic Acids Res..

[32] Fromm B., Domanska D., Høye E., Ovchinnikov V., Kang W., Aparicio-Puerta E., Johansen M., Flatmark K., Mathelier A., Hovig E. (2020). MirGeneDB 2.0: The metazoan microRNA complement. Nucleic Acids Res..

[33] Davis A.P., Grondin C.J., Johnson R.J., Sciaky D., Wiegers J., Wiegers T.C., Mattingly C.J. (2020). Comparative toxicogenomics database (CTD): Update 2021. Nucleic Acids Res..

[34] Lipscomb C.E. (2000). Medical subject headings (MeSH). Bull. Med. Libr. Assoc..

[35] Bello S.M., Shimoyama M., Mitraka E., Laulederkind S.J., Smith C.L., Eppig J.T., Schriml L.M. (2018). Disease ontology: Improving and unifying disease annotations across species. Dis. Models Mech..

[36] Amberger J.S., Hamosh A. (2017). Searching online mendelian inheritance in man (OMIM): A knowledgebase of human genes and genetic phenotypes. Curr. Protoc. Bioinform..

[37] Karagkouni D., Paraskevopoulou M.D., Chatzopoulos S., Vlachos I.S., Tastsoglou S., Kanellos I., Papadimitriou D., Kavakiotis I., Maniou S., Skoufos G. (2018). DIANA-TarBase v8: A decade-long collection of experimentally supported miRNA-gene interactions. Nucleic Acids Res..

[38] Vlachos I.S., Zagganas K., Paraskevopoulou M.D., Georgakilas G., Karagkouni D., Vergoulis T., Dalamagas T., Hatzigeorgiou A.G. (2015). DIANA-miRPath v3.0: Deciphering microRNA function with experimental support. Nucleic Acids Res..

[39] Yang M., Chen J., Su F., Yujie L., Su F., Lin L., Liu Y., Huang J.-D., Song E. (2011). Microvesicles secreted by macrophages shuttle invasion-potentiating microRNAs into breast cancer cells. Mol. Cancer.

[40] Aucher A., Rudnicka D., Davis D.M. (2013). MicroRNAs transfer from human macrophages to hepato-carcinoma cells and inhibit proliferation. J. Immunol..

[41] Pritchard C.C., Kroh E., Wood B., Arroyo J., Dougherty K.J., Miyaji M.M., Tait J.F., Tewari M. (2011). Blood cell origin of circulating microRNAs: A cautionary note for cancer biomarker studies. Cancer Prev. Res..

[42] Kirschner M.B., Edelman J.B., Kao S.C.-H., Vallely M.P., Van Zandwijk N., Reid G. (2013). The impact of hemolysis on cell-free microRNA biomarkers. Front. Genet..

[43] Zhou W., Fong M.Y., Min Y., Somlo G., Liu L., Palomares M.R., Yu Y., Chow A., O’Connor S.T.F., Chin A.R. (2014). Cancer-secreted miR-105 destroys vascular endothelial barriers to promote metastasis. Cancer Cell.

[44] Laterza O.F., Lim L., Garrett-Engele P.W., Vlasakova K., Muniappa N., Tanaka W.K., Johnson J.M., Sina J.F., Fare T.L., Sistare F.D. (2009). Plasma microRNAs as sensitive and specific biomarkers of tissue injury. Clin. Chem..

[45] Yáñez-Mó M., Siljander P.R.-M., Andreu Z., Zavec A.B., Borras F.E., Buzas E.I., Buzas K., Casal E., Cappello F., Carvalho J. (2015). Biological properties of extracellular vesicles and their physiological functions. J. Extracell. Vesicles.

[46] Fehlmann T., Lehallier B., Schaum N., Hahn O., Kahraman M., Li Y., Grammes N., Geffers L., Backes C., Balling R. (2020). Common diseases alter the physiological age-related blood microRNA profile. Nat. Commun..

[47] O’Connell R.M., Rao D., Chaudhuri A.A., Baltimore D. (2010). Physiological and pathological roles for microRNAs in the immune system. Nat. Rev. Immunol..

[48] Chen C.-C., Peng C.-C., Fan P.-C., Chu P.-H., Chang Y.-S., Chang C.-H. (2020). Practical procedures for improving detection of circulating miRNAs in cardiovascular diseases. J. Cardiovasc. Transl. Res..

[49] Marabita F., De Candia P., Torri A., Tegnér J., Abrignani S., Rossi R. (2015). Normalization of circulating microRNA expression data obtained by quantitative real-time RT-PCR. Brief. Bioinform..

[50] Sharma S., Eghbali M. (2014). Influence of sex differences on microRNA gene regulation in disease. Biol. Sex Differ..

[51] Flowers E., Won G.Y., Fukuoka Y. (2015). MicroRNAs associated with exercise and diet: A systematic review. Physiol. Genom..

[52] Rome S. (2015). Use of miRNAs in biofluids as biomarkers in dietary and lifestyle intervention studies. Genes Nutr..

[53] Murillo O., Thistlethwaite W., Rozowsky J., Subramanian S.L., Lucero R., Shah N., Jackson A.R., Srinivasan S., Chung A., Laurent C.D. (2019). ExRNA atlas analysis reveals distinct extracellular RNA cargo types and their carriers present across human biofluids. Cell.

